# Expression of TLR4 Is Upregulated in Patients with Sporadic Acute Stanford Type A Aortic Dissection

**DOI:** 10.1155/2022/3806462

**Published:** 2022-09-23

**Authors:** Xinyi Liu, Ai Zhang, Nianguo Dong, Zhiwen Wang

**Affiliations:** ^1^Department of Cardiovascular Surgery, Institution of Union Hospital, Tongji Medical College, Huazhong University of Science and Technology, Wuhan, Hubei 430022, China; ^2^Department of Rheumatology and Immunology, General Hospital of Central Theater Command, Wuhan, Hubei 430070, China

## Abstract

Sporadic acute Stanford type A aortic dissection (TAAD) is a serious condition that requires urgent treatment to avoid catastrophic consequences. The purpose of the present study was to explore, firstly, whether TLR4-regulated immune signalling molecules were activated in TAAD patients and, secondly, whether TLR4-regulated inflammatory products interleukin-1*β* (IL-1*β*) and CC chemokine ligand 5 (CCL5) could be a promising biomarker for diagnosis in patients with TAAD. Full-thickness ascending aortic wall specimens from TAAD patients (*n* = 12) and control donors (*n* = 12) were examined for the expression of TLR4 and its major signalling molecules, in terms of immunity and inflammation. Blood samples from TAAD (*n* = 49) and control patients (*n* = 53) were collected to detect the circulating plasma cytokine levels of IL-1*β* and CCL5. We demonstrated that expression levels of TLR4 and its downstream signalling cascade molecules were significantly elevated. Furthermore, receiver operating characteristic curve analyses showed that elevated IL-1*β* levels and decreased plasma CCL5 may have diagnostic value for TAAD. In summary, this current study suggests a more generalized pattern of inflammation in TAAD. In addition, TLR4-mediated inflammatory product, such as IL-1*β* and CCL5, could be novel and promising biomarkers with important diagnostic and predictive value in the identification of sporadic TAAD diseases.

## 1. Background

Sporadic acute aortic dissection (AAD) is a catastrophic disease process associated with very high morbidity and mortality. AAD is characterized by medial degeneration with tearing of the intima layer and blood crossing into the artery wall, which leads to the formation of a false lumen within the middle tunica. Depending on the location of the rupture, AAD is classified as acute Stanford type A aortic dissection (TAAD), when the ascending aortic thoracic tract and/or the arch are involved, and Stanford-B aortic dissection, when the descending thoracic aorta and/or aortic abdominal tract are involved [1]. TAAD is the most frequent type of dissection, comprising approximately 75% of all cases, and the mortality can reach 90% if untreated [2]. Unfortunately, at this time, pharmacological agents capable of effectively limiting the progression of TAAD are unavailable, largely because of an incomplete understanding of TAAD pathogenesis.

Increasing evidence suggests that the aorta wall of TAAD has inflammatory cell infiltration and enhanced expression of inflammatory mediators [3]. Among these, it has also been demonstrated that the development of TAAD is tightly linked to an increase in associated inflammatory cells such as macrophages [4]. Toll-like receptor-4 (TLR4) is one of the most well characterized inflammation-related molecules and can identify a number of exogenous and endogenous ligands and initiate the immune inflammatory response [5]. TLR4 expression can be detected on a large array of tissue and cell types, such as aortic wall cells, particularly endothelial cells (ECs) and vascular smooth muscle cells (VSMCs), and it responds to particular damage-related products, activating particular inflammatory [6–8]. TLR4-mediated signalling has been shown to cause inflammatory cell infiltration, the generation of inflammatory mediators, and vascular endothelial cell dysfunction [9]. These are linked to aortic inflammation and remodeling, as well as medial vascular degeneration and aortic dissection [10]. It has been recently reported that the association between TLR4 polymorphisms and AAD susceptibility in the Chinese Han population has been confirmed [8]. However, to date, the expression of TLR4 in TAAD is still unknown. Therefore, the aim of this study was to identify whether the TLR4 pathways, as key inflammatory promoters, are involved in the pathophysiological mechanisms of TAAD and to determine whether their inflammatory products are biomarkers for the early diagnosis of patients with TAAD.

## 2. Materials and Methods

The procedure used combined previous literature methods with those established in our research group [11].

### 2.1. Ethics Statement

This study was approved by the Ethics Committee of Union Hospital. All patients provided signed informed consent before participating in the study. This study conforms to the Code of Ethics of the World Medical Association (Declaration of Helsinki), printed in 1975.

### 2.2. Tissue and Plasma Samples

Appropriate informed written consent was obtained for all procedures. The ascending aortic wall specimens were obtained approximately 3 cm from the aortic annulus of 12 TAAD patients undergoing open surgical treatment. None of the patients had any known genetic syndrome related to aortic disease (e.g., Marfan, Turner, or Ehlers-Danlos syndrome). Patients with a family history of TAAD or with dissection induced by trauma or cardiovascular operations were excluded. The control group consisted of twelve age- and sex-matched nonaortic dissections of aortic specimens obtained from full-thickness thoracic aortic walls from heart or kidney donors provided by the Union Hospital. These control aortic samples were macroscopically normal and devoid of early atheromatous lesions. Immediately after collection, each (thrombus-free) specimen was divided into 2 equivalent pieces. The first half was divided into small (50 mg) full-wall thickness pieces. The second half was carefully dissected into an outer dissected membrane (DM) and an inner DM. Tissue samples were immediately frozen in liquid nitrogen and then stored at −80°C until processing. Demographic and clinical characteristics of the TAAD and control groups are shown in Table 1.

A total of 102 patients were recruited from the Department of the Union Hospital from July 2019 to January 2020. All patients were consecutive patients who suffered from sudden chest pain and underwent computed tomography angiography (CTA). Study participants were excluded from the study if they had one or more of the following conditions: (1) onset time >1 day; (2) autoimmune diseases or infectious diseases that can cause changes in the levels of inflammatory factors; (3) iatrogenic aortic injury, traumatic aortic injury, and drug use; (4) various other heart diseases, such as coronary heart disease, viral myocarditis, cardiomyopathy, cardiac insufficiency, and bicuspid aortic valve; (5) certain genetic syndromes, such as Marfan syndrome, Ehlers-Danlos syndrome, and aortitis; (6) inherited tissue connective diseases; (7) intramural haematoma (IMH) and penetrating aortic ulcer (PAU); and (8) disease accompanied by severe liver and kidney damage. The diagnosis was determined based on CTA results and clinical symptoms of patients by doctors with 18 years of clinical experience, and a total of 102 cases were included. They were separated into two groups based on clinical diagnosis: TAAD (*n* = 49) and non-TAAD (*n* = 53) (Table 2). Each participant provided a fasting blood sample of approximately 5 mL, which was obtained using standardized sterile tubes, and patient cases were gathered. Plasma samples were centrifuged at 3000 r/min for 5 min, separated into 500 *μ*l aliquots, and stored at −80°C. The study was approved by the Institutional Review Board of the Union Hospital.

### 2.3. Western Blot

Briefly, total proteins of each aortic sample were extracted using RIPA lysis buffer (Beyotime Biotechnology, Shanghai, China) containing a protease inhibitor (Thermo Fisher Scientific, Shanghai, China). Protein concentration was quantified by the BCA protein assay kit (Beyotime Biotechnology, Shanghai, China), and an equal number of proteins were separated on 12.5% SDS-PAGE gels. After electrophoresis, the proteins were transferred to polyvinylidene fluoride (PVDF) membranes and incubated with antibodies against TLR4 (Wanleibio, Shenyang, China), myeloid differentiation factor 88 (MyD88, Wanleibio), TNF receptor-associated factor 6 (TRAF-6, Wanleibio), interferon regulatory factor 3 (IRF3, Wanleibio), IL-1*β* (Wanleibio), toll-like receptor-associated activator of interferon (TRIF, Abcam, Cambridge, MA, USA), NF-*κ*B p65 (Abcam), CCL5 (Affinity Biosciences, USA), and *β*-actin (ZSGB-BIO, Beijing, China). The membranes were blocked with 5% fat-free dry milk and then incubated with the appropriate primary antibody for 24 hours at 4°C. The membranes were then treated with a peroxidase-conjugated secondary antibody after three washes (ZSGB-BIO, Beijing, China). Finally, BeyoECL Plus was used to view the immunological complexes (Beyotime Biotechnology, Shanghai, China).

### 2.4. Quantitative Real-Time Polymerase Chain Reaction (qPCR)

TRIzol was used to extract total RNA from the aortic tissues (BioTek, Beijing, China). Moloney murine leukemia virus reverse transcriptase (M-MLV RT) was used to make cDNA from RNA (BioTek, Beijing, China). 2xPower Taq PCR MasterMix was used for real-time PCR (BioTek, Beijing, China). As an endogenous control, the level of *β*-actin mRNA was measured. Table 1 lists the primer sequences for TLR4, MYD88, TRAF-6, NF-κB p65, IL-1, TRIF, IRF3, CCL5, and *β*-actin. For relative quantification, the findings were analyzed using the 2-Ct technique. Each experiment was carried out three times.

### 2.5. Immunohistochemistry (IHC)

IHC was used to determine the expression levels of CD3+ (T lymphocytes), CD20+ (B lymphocytes), and CD68+ cells (macrophages) in aortic wall tissues. Briefly, 6 m formalin-fixed and paraffin-embedded sections were produced, then incubated overnight at 4°C with primary antibodies against CD3, CD20, and CD68 (Abcam, Cambridge, MA, USA), followed by diaminobenzidine staining and counterstaining with haematoxylin. Cover slips were used to mount the sections, which were then inspected under a light microscope.

### 2.6. Cytokine Assay

IL-1 and CCL5 plasma inflammatory cytokine levels were measured using enzyme-linked immunosorbent assays (ELISAs) as directed by the manufacturer (R&D Systems, Beijing, China).

### 2.7. Venous Blood Indexes

On the day of the examination, blood was drawn from each patient, and clinical test results were determined on the same day. An Olympus AU640 Autoanalyzer was used to quantify serum levels of total cholesterol (TC), low-density lipoprotein cholesterol (LDL-C), and high-density lipoprotein cholesterol (HDL-C) using an automated spectrophotometer and an enzymatic colorimetric technique (Olympus, Kobe, Japan). On a Hitachi 7600 Autoanalyzer, several biochemical tests were conducted using the Jaffe kinetic technique (Hitachi, Tokyo).

### 2.8. Statistical Analysis

ANOVA, independent-sample *t*-tests, and Mann–Whitney *U*-tests were used to assess differences between variables. The correlations between plasma IL-1, CCL5, and plasma D-dimer were determined using Spearman's correlation analysis. The diagnostic effects of serum cytokines were evaluated using receiver operating characteristic (ROC) curves, and the associated cut-off points were determined. Multiple logistic regression models with adjustments for potential confounding factors were used to estimate the predictive value of serum IL-1 and CCL5 in assessing TAAD risk. For regularly distributed variables, all data are provided as medians and interquartile ranges, or means and standard deviations. Statistical analysis was performed using SPSS 16.0 (Chicago, IL, USA). *P* < 0.05 was considered significant.

## 3. Results

### 3.1. Expression Levels of TLR4/MyD88 Were Upregulated in the Ascending Aortic Wall in TAAD Patients

**Figure 1:**
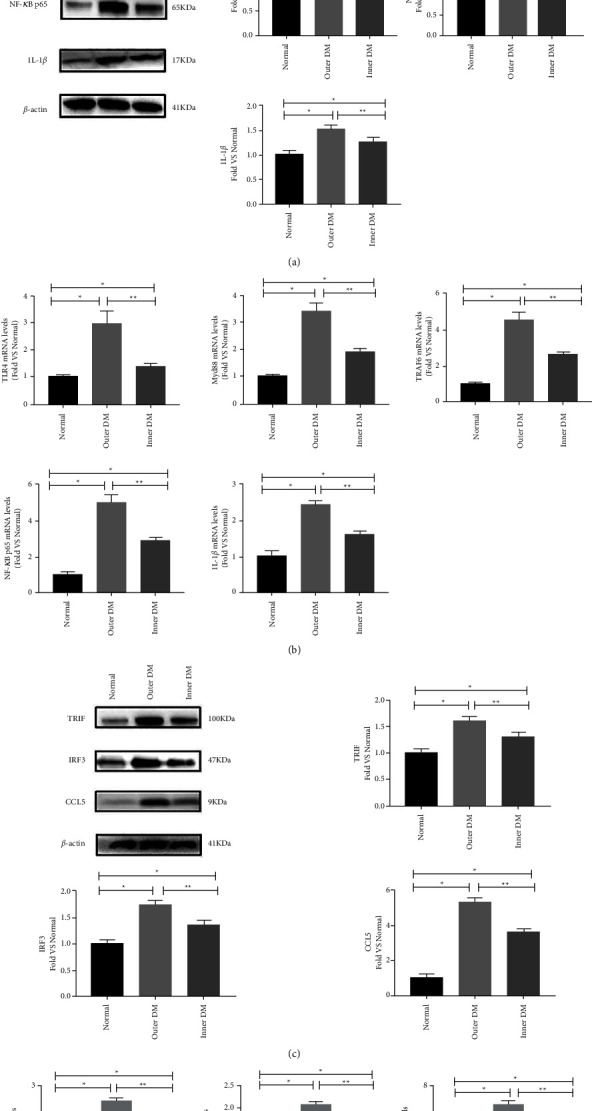
The expression levels of TLR4 signalling pathways were enhanced in TAAD patients. (a, b). Representative results of western blotting and qPCR for TLR4/MyD88 signalling cascade molecule. Expression in TAAD aortic wall tissues and normal control tissues (c, d). Representative results of western blotting and qPCR for TLR4/TRIF signalling cascade molecule expression in TAAD aortic wall tissues and normal control tissues. *β*-Actin was used as the endogenous control. Data are expressed as the means ± SD. *n* = 12. ^*∗*^*P* < 0.05 vs the normal group. ^*∗∗*^*P* < 0.05 vs the outer DM group.

Using western blot analysis, the protein levels of the TLR4 and its Myd88 signalling pathway-related molecule were detected in the TAAD wall excised from the ascending aortic site as well as in the control tissue samples. Following standardization to the expression of *β*-actin, TAAD samples showed significantly increased protein expression levels of TLR4, Myd88, TRAF-6, NF-*κ*B p65, and IL-1*β* compared with control aorta, especially in the outer DM (Figure 1(a)). Moreover, the qPCR analysis also showed similar results (Figure 1(b)).

### 3.2. Expression Levels of TLR4/TRIF Were Upregulated in the Ascending Aortic Wall in TAAD Patients

Following expression analysis of TLR4/MYD88 signalling pathways, the expression of TLR4/TRIF in the TAAD tissue samples was determined at the protein and mRNA levels using western blot and qPCR analysis. The expression levels of TRIF, IRF3 and CCL5 in the outer and inner DMs were markedly increased compared with the normal aorta wall (Figures 1(c) and 1(d)).

### 3.3. Immune Inflammatory Cells in the Ascending Aortic Wall Were Enhanced in TAAD Patients

**Figure 2:**
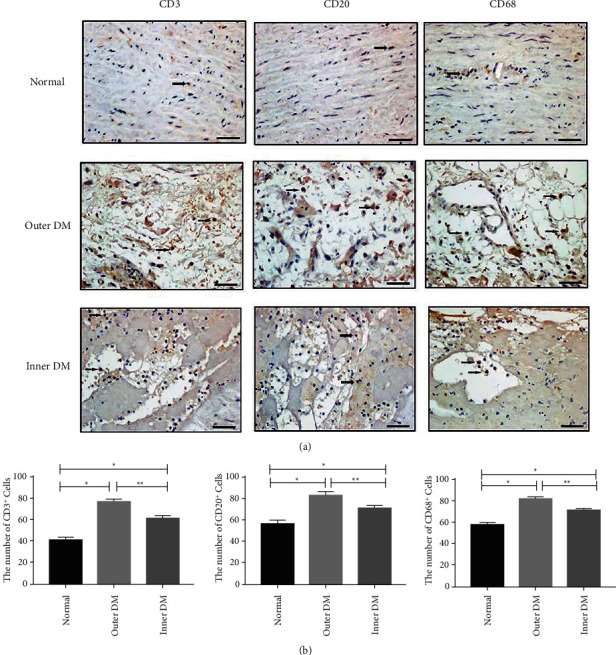
Immune inflammatory cell accumulation in the ascending aorta media in TAAD patients. (a, b) Representative micrographs (a) and quantification (b) of CD3+ (t lymphocytes), CD20+ (b lymphocytes), and CD68+ cells (macrophages) in TAAD aortic wall tissues and normal control tissues. Immunohistochemical staining of ascending aortic wall specimens is shown (×400). Data is expressed as the means ± SD. *n* = 12. ^*∗*^*P* < 0.05vs the normal group. ^*∗∗*^*P* < 0.05 vs the outer DM group.

IHC staining showed that compared with control aorta walls, inflammatory markers for T and B lymphocytes and macrophage levels in the outer and inner DMs were markedly increased (Figures 2(a) and 2(b)). The results indicated recruitment and infiltration of immune inflammatory cells to damaged areas of the ascending aorta wall and induction of an inflammatory reaction.

### 3.4. Clinical Characteristics of Patients and Comparisons between Patients with and Those without TAAD

Table 2 shows the characteristics of 102 study participants, among whom 49 were diagnosed with TAAD. Various clinical parameters in the study participants were compared between those with and those without TAAD. TAAD patients had a more pronounced plasma inflammatory response [12.24 (9.26 − 13.86) × 10^9^/L *vs* 7.36 (5.97 − 10.17) × 10^9^/L] and a significantly higher prevalence of hypertension [35 (71.4%)] than the TAAD-free controls [21 (39.6%)]. Furthermore, TAAD patients showed a tendency towards having a higher serum D-dimer than TAAD-free controls [5.41 (3.12 − 8.88) mg/L vs 0.98 (0.52 − 1.26) mg/L]. Moreover, the above indicators were significantly differentially expressed in TAAD patients compared to TAAD-free controls (*P* < 0.001). The serum level of IL-1*β* was 3.90 (3.20 − 4.87) pg/mL, which was significantly higher than the reported value of 2.94 (2.05 − 3.77) pg/mL in subjects without TAAD, whereas the serum levels of CCL5 were lower than those in subjects without TAAD (*P* < 0.001).

### 3.5. Correlations of Serum IL-1*β* and CCL5 with TAAD-Related Circulating Biomarkers

**Figure 3:**
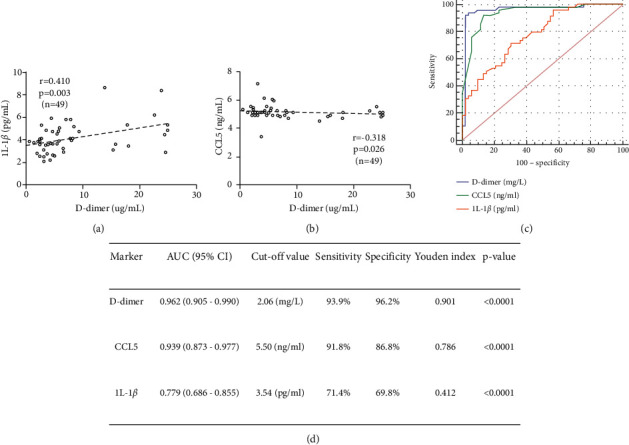
Correlations among IL-1*β* levels, CCL5 levels, and D-dimer levels and their diagnostic value in TAAD. (a). Correlation between IL-1*β* levels and D-dimer levels. (b). Correlation between CCL5 levels and D-dimer levels. (c). ROC curves of IL-1*β*, CCL5, and D-dimer for the diagnostic value of TAAD. Data are expressed as the means ± SD.*P* values were calculated using Spearman's rank correlation. (d). Detailed digital results of the diagnostic value of the ROC curves of IL-1*β*, CCL5, and D-dimer.

Many studies have shown that plasma D-dimer levels have a significant positive correlation with the incidence of TAAD and thus may be an effective predictor of the incidence of TAAD. Therefore, we further analyzed the association between plasma IL-1*β* and CCL5 levels and D-dimer levels in acute TAAD patients by Spearman's correlation analysis. The results showed that IL-1*β* levels were positively correlated with D-dimer, whereas the serum levels of CCL5 were negatively correlated with D-dimer (Figures 3(a) and 3(b)). Multiple logistic regression analysis was employed to examine whether serum IL-1*β* or CCL5 was independently and significantly associated with the presence of TAAD (Table 3). Model 1, which included IL-1*β*, in addition to sex, age, smoking history, presence/absence of hypertension, D-dimer, white blood cells (WBCs), TC, LDL-C, and HDL-C as independent variables, showed that IL-1*β*, in addition to D-dimer, was a significant and independent factor associated with the presence of TAAD (odds ratio: 2.04, 95% confidence interval (CI): 1.13–3.71, *P*=0.019). Model 2, in which IL-1*β* was replaced with CCL5, demonstrated that CCL5 was significantly associated with the presence of TAAD (odds ratio: 0.031, 95% CI: 0.01–0.18, *P* < 0.001).

### 3.6. Diagnostic and Predictive Value of Serum IL-1*β*, CCL5, and D-Dimer in TAAD Diseases

The diagnostic accuracy of IL-1*β*, CCL5 and D-dimer levels in discriminating TAAD diseases is shown in Figure 3(c). The optimal cut-off value of D-dimer was 2.06 (mg/l), with an area under the curve (AUC) of 0.962 (95% CI: 0.905–0.990), a sensitivity of 93.9%, and a specificity of 96.2%. The AUC values (95% CI) for CCL5 and IL-1*β* were 0.939 (95% CI: 0.873–0.977) and 0.779 (95% CI: 0.686–0.855), with corresponding optimal cut-off points of 5.50 (ng/ml) and 3.54 (pg/ml), which were associated with sensitivities of 91.8% and 71.4% and specificities of 86.8% and 69.8%, respectively (Figure 3(d)). The ROC analysis results revealed that these three cytokines may have diagnostic value in TAAD; D-dimer had the best ROC for diagnosis, followed by CCL5 and finally IL-1*β*.

## 4. Discussion

The pathogenesis of TAAD and, in particular, identification of circulatory biomarkers, remains a focus of intense research interest. TLR4 has been reported to have a similar regulating role in the immune response and to provide information about the systemic status associated with inflammatory conditions.

Previous research has suggested that TLR4 may have a role in the development of thoracic aortic aneurysms (TAA), whereas fewer reports have investigated the role of TLR4 in TAAD. Although there are still some distinctions in the pathogenesis of the two, TAA and TAAD are both intimately associated to local and systemic immune inflammatory responses. Therefore, the potential role of TLR4 in enhancing the immune inflammation that leads to TAAD needs to be clarified.

First, we found that TLR4 was overexpressed in the aortic tissues of TAAD. These observations suggest that increased expression of TLR4 may be related to TAAD. There are two adapter molecules essential for TLR4 signalling: MyD88 and TRIF. MyD88-mediated signalling promotes early-phase activation of NF-*κ*B, which controls the production of tumour necrosis factor-a (TNF- a), interleukin-6 (IL-6), and IL-1*β* [12, 13]. The TRIF-dependent pathway engages IRF3 and drives the expression of genes encoding type I interferons and some cytokines, such as the chemokine CCL5. Late-phase activation of NF-*κ*B is also induced in this pathway [14, 15]. To explore the significance of TLR4 upregulation in TAAD, related molecules in both pathways were analyzed. We found that TLR4 and its downstream signalling cascade molecules were overexpressed in the aortic tissues of TAAD. Our results also demonstrated a pronounced accumulation of macrophages and B and T lymphocytes in the aorta media in TAAD patients, indicative of an immune inflammatory response. Interestingly, recent studies have demonstrated that activated macrophages are able to release and regulate the activity of proinflammatory cytokines (IL-1*β* and CCL5), which play a crucial role in maintaining aortic wall inflammation, leading to matrix degradation [16]. Furthermore, previous studies have demonstrated that macrophages produce endogenous ligands that can bind to several receptors, including receptors for advanced glycation end products (RAGE), TLR-2, and TLR-4, thereby triggering cell signalling pathways involving mitogen-activated protein kinase (MAPK), NF-*κ*B, or phosphatidylinositol 3 kinase (PI3K)/protein kinase B (Akt) to mediate cell migration, activation, proliferation, and differentiation [17–20]. Thus, these data, combined with our preliminary findings, suggest that activated macrophages induce the over-activation of TLR-4 signalling pathways, which release so-called inflammatory mediators, such as IL-1*β* and CCL5. This hypothesis suggests that the early stages of TAAD are associated with a predominant infiltration of macrophages whose characteristics are induced by TLR4 and that probably enhance vascular inflammation.

In light of the above findings, we further investigated whether the TLR4-regulated inflammatory products, IL-1*β* and CCL5, are potential diagnostic markers and therapeutic targets for TAAD. First, we identified elevated plasma IL-1*β* and decreased plasma CCL5 as independent risk markers in TAAD. Epidemiologic studies, thus far performed mainly in patients with aortic dissection (AD), have established the notion that serum D-dimer is positively and independently associated with predictors for AD risks [21]. Thus, we assessed the relationship between serum D-dimer and TAAD-related circulating biomarkers and found modest associations of serum IL-1*β* and CCL5 levels with plasma D-dimer in TAAD patients. Moreover, we evaluated the diagnostic and predictive features of circulating biomarkers for identifying TAAD, which may be helpful for making a reasonable clinical decision in diagnostic and screening procedures. Based on ROC curves, elevated IL-1*β* and decreased CCL5 levels suggested a high specificity and sensitivity in recognizing TAAD, but it was less than that of D-dimer. Indeed, a significant relationship between the pathogenesis of AAA and serum IL-1*β* and CCL5 levels has been reported, indicating that aortic aneurysm formation and progression could be blocked by genetic or pharmacological inhibition of IL-1*β* or CCL5 [22, 23]. However, the relationships between the above two cytokines and TAAD are still unclear. In this current study, we measured inflammatory factors in tissues and plasma and simulated confounded interactions between inflammatory factors and other demographic and clinical confounders and identified elevated plasma IL-1*β* and decreased plasma CCL5 as independent risk markers in TAAD.

The most interesting association observed in this study was CCL5, which showed differential expression in both aortic tissue and plasma. CCL5 is a CC chemokine family T-cell mitogen that exerts a broad spectrum of effects on inflammatory responses. However, CCL5 is also produced by other cells, including macrophages, T-cells, dendritic cells, smooth muscle cells, and platelets (PLTs), and has been shown to be associated with AAA risk [24]. Similar to our current study, CCL5 mRNA and protein levels have been previously reported to be elevated in AAA aortic wall tissue [25]. In contrast to tissue expression, we observed significantly lower plasma CCL5 in TAAD patients than in the controls. It is also worth noting that because CCL5 is synthesized, stored in platelets, and then released upon platelet activation, TAAD has been previously linked to reduced circulating PLT counts and increased PLT activation [26], both of which may partly explain the increased localization (TAAD wall) and decreased circulating levels of PLT-associated factors, such as CCL5.

This study revealed numerous immune infiltrates composed of macrophages and lymphocytes in the ascending aortic wall in TAAD patients, compared with few infiltrates in control patients. Interestingly, the infiltration of inflammatory cells was particularly high in the outer DM from the TAAD patient samples. Moreover, the activation of the TLR4 signalling pathway was more pronounced in the outer DM. Currently, identifying the initialization and propagation of vascular inflammation has been a controversial focus [27]. The results of our experiments provided emerging data to support the “outside-in” hypothesis, in which the process of inflammatory cytokines mediated by local immune responses initiates in the adventitia and progresses inward towards the intima [28–30].

The current study had several limitations that should be mentioned. First, due to the limitations of related experiments, we did not verify the regulatory mode between TLR4 and IL-1*β*/CCL5. Second, due to the current lack of relevant data, we were unable to accurately account for changes in serum IL-1*β* and CCL5 levels in 12 patients compared with controls. Moreover, both the aortic sample and blood sample sizes are small, and more patients are needed. Finally, the conclusions were based solely on human aortic wall specimens. Despite these limitations, these preliminary results will provide clues to the molecular mechanisms involved in TAAD, which should be pursued in future investigations.

## 5. Conclusions

**Figure 4:**
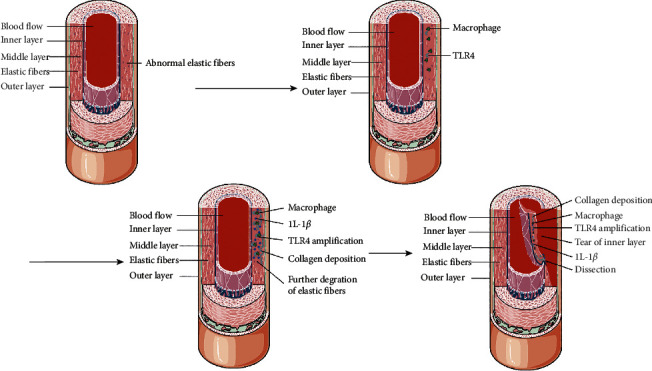
Based on our analysis of TAAD patients, we hypothesized the presence of abnormal elastic fibers in the medial layer of the vessel wall. It allows macrophages to migrate to the site of aortic injury where macrophage ligands bind to TLR4 receptors, further aggravating the degradation of elastic fibers. Activated macrophages involved in exacerbated local inflammatory responses mediate TLR4 expansion, increase the release of inflammatory factors such as IL-1*β*, and induce collagen deposition, resulting in impaired aortic wall compliance.

In conclusion, our research results and together with the results from some previous studies, in the specific case of sporadic TAAD, we propose a sequential “two-hit” model of vascular inflammation involving initial vascular injury followed by recruitment of macrophages to injured sites in the aorta. Activated macrophages produce amplification of IL-1*β* and CCL5 expression that converges on common, pathogenic TLR4 signal transducers and activation of the NF-*κ*B p65 signalling pathway. This pathway stimulates the effector functions of macrophages and enhances inflammatory factor expression, ultimately resulting in deterioration of the vascular wall structural integrity (Figure 4). Furthermore, our evidence suggests the possible migration of inflammatory cells from the adventitial side of the vessel towards the intima. Ultimately, TLR4-mediated inflammatory products, such as IL-1*β* and CCL5, may be novel and promising biomarkers with important diagnostic and predictive value in the identification of TAAD diseases.

## Figures and Tables

**Table 1:** Aortic tissue population demographic.

	Controls, *n* = 12	TAAD, *n* = 12	*P* value
Age (years)	46 (31–52)	49 (43–57)	0.381
Male sex (%)	8 (66.7%)	8 (66.7%)	1.0
Smoking (*n*[%])	3 (25.0%)	4 (33.3%)	0.628
Hypertension *n*[%])	2 (16.7%)	8 (66.7%)	<0.001
D-Dimer (mg/L)	0.92 (0.58–1.21)	5.82 (3.61–8.65)	<0.001
WBC (×10^9^/L)	7.45 (5.78–11.23)	12.85 (10.4–13.38)	<0.001
TC (mmol/L)	4.19 ± 0.32	4.24 ± 0.21	0.85
LDL-C (mol/L)	2.32 (2.18–3.21)	2.49 (2.31–3.27)	0.72
HDL-C (mol/L)	1.23 (1.05–1.28)	1.15 (1.02–1.31)	0.63
CREA (umol/L)	76 (51–86)	93 (67–108)	0.56

TAAD: acute Stanford type A aortic dissection; WBC: white blood cell; TC: total cholesterol; HDL-C: high-density lipoprotein cholesterol; LDL-C: low-density lipoprotein cholesterol; CREA: creatinine.

**Table 2:** Clinical and biochemical characteristics of the study participants.

	All subjects	With TAAD	Without TAAD	*P* value
Patients (*n*)	102	49	53	
Male sex (%)	60 (58.8%)	29 (59.2%)	31 (58.5%)	0.94
Age (years)	50 (46–58)	51 (43–60)	49 (47–58)	0.85
Smoking (*n*[%])	32 (31.4%)	17 (34.7%)	15 (28.3%)	0.73
Hypertension (*n*[%])	56 (54.9%)	35 (71.4%)	21 (39.6%)	0.001
D-dimer (mg/L)	1.91 (0.89–5.42)	5.41 (3.12–8.88)	0.98 (0.52–1.26)	<0.001
CCL5 (ng/mL)	5.50 (5.10–6.70)	5.10 (4.90–5.25)	6.70 (6.10–7.10)	<0.001
IL-1*β* (pg/mL)	3.56 (2.49–4.12)	3.90 (3.20–4.87)	2.94 (2.05–3.77)	<0.001
WBC (×10^9^/L)	9.45 (6.74–12.6)	12.24 (9.26–13.86)	7.36 (5.97–10.17)	<0.001
TC (mmol/L)	4.27 ± 0.08	4.26 ± 0.13	4.27 ± 0.11	0.99
LDL-C (mol/L)	2.65 (2.26–3.15)	2.67 (2.56–3.13)	2.45 (2.31–3.03)	0.82
HDL-C (mol/L)	1.17 (1.05–1.38)	1.14 (1.03–1.38)	1.21 (1.08–1.39)	0.44

TAAD: acute Stanford type A aortic dissection; WBC: white blood cell; TC: total cholesterol; HDL-C: high-density lipoprotein cholesterol; LDL-C: low-density lipoprotein cholesterol; IL-1*β*: interleukin-1*β*; CCL5: CC chemokine ligand 5.

**Table 3:** Multiple logistic regression analysis of factors associated with TAAD.

	Model 1			Model 2		
	OR	95% CI	*P* value	OR	95% CI	*P* value
Male sex (%)	2.58	0.73–9.05	0.14	3.23	0.53–19.8	0.21
Age (years)	0.98	0.91–1.05	0.55	0.95	0.85–1.06	0.35
Smoking (*n*[%])	1.04	0.84–1.21	0.64	1.08	0.90–1.27	0.51
Hypertension (*n*[%])	2.65	0.80–8.74	0.11	2.34	0.43–12.8	0.33
D-dimer (mg/L)	1.66	1.20–2.29	0.002	1.34	1.04–1.73	0.02
WBC (×10^9^/L)	1.15	0.97–1.37	0.12	1.06	0.82–1.38	0.65
TC (mmol/L)	1.21	0.58–2.47	0.62	1.47	0.55–3.93	0.44
LDL-C (mol/L)	0.78	0.23–2.61	0.68	0.95	0.65–1.41	0.81
HDL-C (mol/L)	0.64	0.05–7.70	0.73	0.48	0.01–18.3	0.69
CCL5 (ng/ml)				0.031	0.005–0.179	<0.001

IL-1*β* (pg/ml)	2.04	1.13–3.71	0.019			
	*R* ^2^ = 0.65, *P* < 0.001			*R* ^2^ = 0.83, *P* < 0.001		

TAAD: acute Stanford type A aortic dissection; OR: odds ratio; 95% CI: 95% confidence intervals; WBC: white blood cell; TC: total cholesterol; HDL-C: high-density lipoprotein cholesterol; LDL-C: low-density lipoprotein cholesterol; IL-1*β*: interleukin-1*β*; CCL5: CC chemokine ligand 5.

## Data Availability

The data used to support the findings of this study are available from the corresponding author upon request.
